# 

**Published:** 1913-11-15

**Authors:** 


### ANNOUNCEMENTS.

### National Dental Association.

#### ADVANCES DATE OF 1914 MEETING ONE WEEK.

At the urgent request of the Local Committee of Arrangements at Rochester, the Trustees of the National Dental Association have advanced the date of the next meeting one week; therefore, the 18th Annual Session will be held in

Rochester, N. Y., July 7, 8, 9, 10, instead of July 14, 15, 16, 17 as originally selected. The officers, the local committee and all other committees are going to put forth every effort to make this meeting which is the first under the re-organization, the best in the history of the Association, and we feel confident that our increased membership and interest in our Association will prove a decided advantage in many ways.

HOMER C. BROWN, Pres.,
Columbus, Ohio.
OTTO U. KING, Gen Secy.,
Huntington, Ind.

### BACK COPIES OF THE PROCEEDINGS OF THE NATIONAL DENTAL ASSOCIATION.

There are a few copies of the '07, '08, '09, '10 and '11 "Transactions of the National Dental Association" in the possession of Dr. Arthur Melendy. These copies, while they last, may be secured by Libraries and other educational institutions and members of the N. D. A. by sending thirty cents per copy (to cover postage) to Dr. Arthur R. Melendy, Holston Bank Building, Knoxville, Tenn.

OTTO U. KING, Gen. Sec.,
Huntington, Ind.

### OHIO STATE DENTAL SOCIETY.

The forty-eighth annual meeting of the Ohio State Dental Society will be in Memorial Hall, Toledo, December 2, 3 and 4, 1913.

The program of papers and clinics carries the names of men of world-wide reputation.

At a "Health and Science" conference, to be held Wednesday evening, we expect to have with us Gov. Cox, Dr. Vaughn, President of the American Medical Association, Dr. Flexner of the Rockefeller Institute, Dr. E. C. Kirk, editor of the *Cosmos*, Dr. McCampbell, Secretary of the State Board of Health and the President of the National Dental Association, Dr. H. C. Brown.

Mark the dates off, make your hotel reservations early and come to the greatest meeting of this Society ever held.

F. R. CHAPMAN, Secy.

### NEW JERSEY BOARD OF EXAMINERS.

The New Jersey State Board of Dental Examiners will hold their regular annual meeting and examination in the Assembly Chamber of the State House at Trenton, N. J., December 1, 2 and 3, 1913.

After January 1, 1914, all applicants for a license to practice dentistry in New Jersey "shall present to said Board a certificate from the Superintendent of Public Instruction showing that before entering a dental college, he or she has obtained an academic education consisting of a four year's course of study in an approved high school (public or private), or the equivalent thereof." A bridge, consisting of three or more teeth, exclusive of abutments, and one Richmond Crown will be accepted as a practical test in prosthetic dentistry, in place of a full set of teeth soldered upon a gold or coin silver plate hitherto required.

Applications must be filed at least ten days prior to date set for examination. For further particulars, apply to

ALPHONSO IRWIN, D.D.S.,
Secretary,
425 Cooper St., Camden, N. J.

### INSTITUTE OF DENTAL PEDAGOGICS.

The next annual meeting of the Institute of Dental Pedagogics will be held in Buffalo, N. Y., January 27, 28, 29, 1914. The Executive Committee is planning to present an exceptionally interesting program which no dental teacher can afford to miss.

J. F. BIDDLE, *Secretary*,
Pittsburgh, Pa.

